# Existence and Uniqueness of Positive Solution for Discrete Multipoint Boundary Value Problems

**DOI:** 10.1155/2014/531978

**Published:** 2014-10-28

**Authors:** Huili Ma, Huifang Ma

**Affiliations:** Department of Mathematics, Northwest Normal University, Lanzhou 730070, China

## Abstract

It is expected in this paper to investigate the existence and uniqueness of positive solution for the following difference equation: −Δ^2^ *u*(*t* − 1) = *f*(*t*, *u*(*t*)) + *g*(*t*, *u*(*t*)), *t* ∈ *ℤ*_1,  *T*_, subject to boundary conditions either *u*(0) − *β*Δ*u*(0) = 0, *u*(*T* + 1) = *αu*(*η*) or Δ*u*(0) = 0, *u*(*T* + 1) = *αu*(*η*), where 0 < *α* < 1, *β* > 0,  and *η* ∈ *ℤ*_2,*T*−1_. The proof of the main result is based upon a fixed point theorem of a sum operator. It is expected in this paper not only to establish existence and uniqueness of positive solution, but also to show a way to construct a series to approximate it by iteration.

## 1. Introduction

Let *T* > 1 be an integer; *ℤ*_*a*,*b*_ : = {*a*, *a* + 1,…, *b*}, where *a*, *b* are positive integers. Difference equation appears as natural descriptions of observed evolution phenomena because most measurements of time evolving variables are discrete and so it arises in many physical problems, as nonlinear elasticity theory or mechanics, and engineering topics. In recent years, the study of positive solutions for discrete boundary value problems has attracted considerable attention, but most research dealt with two-point boundary value problem; see [1–4] and the references therein. For multipoint boundary value problem, there appeared a small sample of work related to the existence of positive solution; we refer the reader to [5–7]. However all of them do not address the problem with the uniqueness of positive solution.

In this paper, we consider the existence uniqueness and positive solutions for difference equation

(1) $$\begin{array}{c} -\Delta^{2}u\left(t-1\right)=f\left(t,u\left(t\right)\right)+g\left(t,u\left(t\right)\right),\;t\in Z_{1,T}, \end{array}$$

subject to boundary conditions

(2) $$\begin{array}{c} \left(\text{i}\right)\;\;u\left(0\right)-\beta \Delta u\left(0\right)=0,\;\;u\left(T+1\right)=\alpha u\left(\eta \right), \end{array}$$

or

(3) $$\begin{array}{c} \left(\text{ii}\right)\;\;\Delta u\left(0\right)=0,\;\;u\left(T+1\right)=\alpha u\left(\eta \right), \end{array}$$

where 0 < *α* < 1, *β* > 0, and *η* ∈ *ℤ*_2,*T*−1_.

Let *E* = *C*(*ℤ*_0,*T*+1_, *ℝ*) denote the class of real valued functions *ω* on *ℤ*_0,*T*+1_ with norm ||*ω*|| = max⁡_*k*∈*ℤ*_0,*T*+1__ | *ω*(*k*)|. Observe that *E* is a Banach space. Set *P* = {*u* ∈ *E* : *u*(*t*) ≥ 0, *t* ∈ *ℤ*_0,*T*+1_} to be the normal cone in *E* with the normality constant 1. For *u*, *v* ∈ *E*, the notation *u* ~ *v* means that there exist *λ* > 0 and *μ* > 0 such that *λv* ≤ *u* ≤ *μv*. Clearly, ~ is an equivalence relation. Given *h* > *θ*, we denote by *P*_*h*_ the set *P*_*h*_ = {*x* ∈ *E*∣*x* ~ *h*}.

**Remark 1 .**

As suggested by the notation, by equipping *ℤ*_0,*T*+1_ with the discrete topology, every *ω* ∈ *C*(*ℤ*_0,*T*+1_) is continuous.

**Definition 2 (see [8]).**

Let *D* = *P* or *D* = *P* ^0^ and let *γ* be a real number with 0 ≤ *γ* < 1. An operator *A* : *P* → *P* is said to be *γ*-concave if it satisfies

(4) $$\begin{array}{c} A\left(tx\right)\geq t^{\gamma }Ax,\;t\in \left(0,1\right),\;x\in D. \end{array}$$

**Definition 3 (see [8]).**

An operator *A* : *E* → *E* is said to be homogeneous if it satisfies

(5) $$\begin{array}{c} A\left(tx\right)=tAx,\;t>0,\;x\in E. \end{array}$$

An operator *A* : *P* → *P* is said to be subhomogeneous if it satisfies

(6) $$\begin{array}{c} A\left(tx\right)\geq tAx,\;t>0,\;x\in P. \end{array}$$

The main tool of this paper is the following fixed point theorem.

**Theorem 4 (see [9]).**

Let *P* be a normal cone in a real Banach space *E*, *A* : *P* → *P* an increasing *γ*-concave operator, and *B* : *P* → *P* an increasing subhomogeneous operator. Assume that

 (i) there is *h* > 0 such that *Ah* ∈ *P*_*h*_ and *Bh* ∈ *P*_*h*_;
 (ii) there exists a constant *δ*_0_ > 0 such that, for any *x* ∈ *P*, *Ax* ≥ *δ*_0_ *Bx*.

Then the operator equation *Ax* + *Bx* = *x* has a unique solution *x* ^*^ ∈ *P*_*h*_. Moreover, constructing successively the sequence *y*_*n*_ = *Ay*_*n*−1_ + *By*_*n*−1_, *n* = 1,2,…, for any initial value *y*_0_ ∈ *P*_*h*_, one has *y* → *x* ^*^ as *n* → *∞*.

**Remark 5 .**

When *B* is a null operator, Theorem 4 also holds.

## 2. Positive and Uniqueness of Solutions to BVP (1)-(2)

In this section, we will apply Theorem 4 to study the existence and uniqueness of positive solution for (1)-(2).

**Lemma 6 (see [6]).**

If *T* ∈ {4, 5,…}, *η* ∈ *ℤ*_2,*T*−1_, and *α*, *β* ∈ *ℝ* are real numbers with *β* ≠ −1 and (*T* + 1 − *αη*) + *β*(1 − *α*) ≠ 0, for any *y* defined in *ℤ*_0,*T*+1_, the nonlocal boundary value problem

(7) $$\begin{array}{c} -\Delta^{2}u\left(t-1\right)=y\left(t\right),\;t\in Z_{1,T},\\ u\left(0\right)-\beta \Delta u\left(0\right)=0,\;\;u\left(T+1\right)=\alpha u\left(\eta \right) \end{array}$$

has a unique solution

(8) $$\begin{array}{c} u\left(t\right)=\overset{T}{\underset{s=1}{\sum }}G\left(t,s\right)y\left(s\right), \end{array}$$

where

(9) G(t,s)={(s+β)[T+1−t−α(η−t)](T+1−αη)+β(1−α),s<min⁡{t,η},(T+1−t)(s+β)+α(η+β)(t−s)(T+1−αη)+β(1−α),η≤s<t,(t+β)[T+1−s−α(η−s)](T+1−αη)+β(1−α),t≤s<η,(T+1−s)(t+β)(T+1−αη)+β(1−α),max⁡{t,η}≤s.

**Lemma 7 .**

For *t* ∈ *ℤ*_0,*T*+1_, *s* ∈ *ℤ*_1,*T*_, the Green function *G*(*t*, *s*) in Lemma 6 has the following properties:

 (i) *G*(*t*, *s*) > 0, *t* ∈ *ℤ*_0,*T*+1_, *s* ∈ *ℤ*_1,*T*_;
 (ii) *G*(*t*, *s*) ≤ *r*(*s*)*h*(*t*), *t* ∈ *ℤ*_0,*T*+1_, *s* ∈ *ℤ*_1,*T*_, where
  (10) $$\begin{array}{c} r\left(s\right)=\frac{\left(T+1-s\right)+\alpha T\left(\eta +\beta \right)}{\left(T+1-\alpha \eta \right)+\beta \left(1-\alpha \right)},\;\;h\left(t\right)=t+\beta ; \end{array}$$
 (iii) for *s* ≥ *t*, *G*(*t*, *s*) = *p*(*s*)*h*(*t*), *t* ∈ *ℤ*_0,*T*+1_, *s* ∈ *ℤ*_1,*T*_, where
  (11) $$\begin{array}{c} p\left(s\right)=\{\begin{array}{cc} \frac{T+1-s-\alpha \left(\eta -s\right)}{\left(T+1-\alpha \eta \right)+\beta \left(1-\alpha \right)}, & s<\eta ,\\ \frac{T+1-s}{\left(T+1-\alpha \eta \right)+\beta \left(1-\alpha \right)}, & \eta \leq s. \end{array} \end{array}$$

**Proof**

Since (i) and (iii) are obvious, here we just prove (ii). For *s* < *t*, *s* < *η*, notice that *t* − *η* < *t* − *s*,

(12) G(t,s)≤(s+β)(T+1−t)+α(t−s)(η+β)(T+1−αη)+β(1−α)≤(t+β)(T+1−s)+α(T+1−s)(η+β)(T+1−αη)+β(1−α)≤(t+β)[(T+1−s)+αT(η+β)](T+1−αη)+β(1−α).

For *η* ≤ *s* < *t*,

(13) G(t,s)≤(s+β)(T+1−t)+αT(η+β)(T+1−αη)+β(1−α)≤(t+β)(T+1−s)+αT(η+β)(T+1−αη)+β(1−α)≤(t+β)[(T+1−s)+αT(η+β)](T+1−αη)+β(1−α).

For *t* ≤ *s* < *η*,

(14) G(t,s)≤(t+β)(T+1−s)(T+1−αη)+β(1−α)≤(t+β)[(T+1−s)+αT(η+β)](T+1−αη)+β(1−α).

For *t* ≤ *s*, *η* ≤ *s*,

(15) G(t,s)=(t+β)(T+1−s)(T+1−αη)+β(1−α)≤(t+β)[(T+1−s)+αT(η+β)](T+1−αη)+β(1−α).

That is, for any *t* ∈ *ℤ*_0,*T*+1_ and *s* ∈ *ℤ*_1,*T*_, *G*(*t*, *s*) ≤ *r*(*s*)*h*(*t*).

**Theorem 8 .**

Assume that

 (A1) *f*, *g* : *ℤ*_0,*T*+1_ × [0, *∞*)→[0, *∞*) are continuous and increasing with respect to the second variable, *g*(*T*, 0) ≠ 0;
 (A2) *g*(*t*, *λx*) ≥ *λg*(*t*, *x*) for *λ* ∈ (0, 1),  *t* ∈ *ℤ*_0,*T*+1_, *x* ∈ [0, *∞*) and there exists a constant *γ* ∈ (0, 1) such that *f*(*t*, *λx*) ≥ *λ* ^*γ*^ *f*(*t*, *x*) for *λ* ∈ (0, 1), *t* ∈ *ℤ*_0,*T*+1_, *x* ∈ [0, *∞*);
 (A3) there exists a constant *δ*_0_ > 0 such that *f*(*t*, *x*) ≥ *δ*_0_ *g*(*t*, *x*), *t* ∈ *ℤ*_0,*T*+1_, *x* ∈ [0, *∞*).

The problem (1)-(2) has a unique positive solution *u* ^*^ ∈ *P*_*h*_, where *h*(*t*) = *t* + *β*, *t* ∈ *ℤ*_0,*T*+1_. Moreover, for any initial value *u*_0_ ∈ *P*_*h*_, constructing successively the sequence

(16) un+1(t)=∑s=1TG(t,s)[f(s,un(s))+g(s,un(s))],iiiiiiiiiiiiiiiiiiiiiiiiiiiiiiiiiiiiiiiiiiiin=0,1,2,…,

we have *u*_*n*_(*t*) → *u* ^*^(*t*) as *n* → *∞*, where *G*(*t*, *s*) is given as (9).

**Proof**

Define two operators *A* : *P* → *E* and *B* : *P* → *E* by

(17) $$\begin{array}{c} \left(Au\right)\left(t\right)=\overset{T}{\underset{s=1}{\sum }}G\left(t,s\right)f\left(s,u\left(s\right)\right),\\ \left(Bu\right)\left(t\right)=\overset{T}{\underset{s=1}{\sum }}G\left(t,s\right)g\left(s,u\left(s\right)\right). \end{array}$$

It is easy to see that *u* is a solution of (1)-(2) if and only if *u* = *Au* + *Bu*. From (A2) and Lemma 7, we know that *A* : *P* → *P* and *B* : *P* → *P*. In the sequel we check that *A*, *B* satisfy all assumptions of Theorem 4.

Firstly, we prove that *A*, *B* are two increasing operators. In fact, by (A1) and Lemma 6, for *u*, *v* ∈ *P* with *u* ≥ *v*, we know that *u*(*t*) ≥ *v*(*t*), *t* ∈ *ℤ*_0,*T*+1_, and obtain

(18) (Au)(t)=∑s=1TG(t,s)f(s,u(s))≥∑s=1TG(t,s)f(s,v(s))=(Av)(t).

Similarly, *Bu* ≥ *Bv*.

Next we show that *A* is a *γ*-concave operator and *B* is a subhomogeneous operator. In fact, for any *λ* ∈ (0, 1) and *u* ∈ *P*, from (A2), we know that

(19) (Aλu)(t)=∑s=1TG(t,s)f(s,λu(s))≥λγ∑s=1TG(t,s)f(s,u(s))=λγ(Au)(t).

That is, *A* is a *γ*-concave operator. At the same time, for any *λ* ∈ (0, 1) and *u* ∈ *P*, from (A2), we get

(20) (Bλu)(t)=∑s=1TG(t,s)g(s,λu(s))≥λ∑s=1TG(t,s)g(s,u(s))=λ(Bu)(t).

So *B* is subhomogeneous.

Now we show that *Ah* ∈ *P*_*h*_ and *Bh* ∈ *P*_*h*_. From (A3) and Lemma 7,

(21) (Ah)(t)=∑s=1TG(t,s)f(s,h(s))≥∑s=tTG(t,s)f(s,h(s))=h(t)∑s=tTp(s)f(s,s+β)≥h(t)∑s=tTp(s)f(s,0)≥δ0h(t)∑s=tTp(s)g(s,0)≥δ0p(T)g(T,0)h(t):=l1h(t),(Ah)(t)=∑s=1TG(t,s)f(s,h(s))≤h(t)∑s=1Tr(s)f(s,h(s))≤h(t)∑s=1Tr(s)f(s,s+β):=l2h(t),

where *l*_1_ = *δ*_0_ *p*(*T*)*g*(*T*, 0) > 0 and *l*_2_ = ∑_*s*=1_^*T*^ *r*(*s*)*f*(*s*, *s* + *β*). Hence we have *l*_1_ *h*(*t*)≤(*Ah*)(*t*) ≤ *l*_2_ *h*(*t*), *t* ∈ *ℤ*_0,*T*+1_; that is, *Ah* ∈ *P*_*h*_. We can similarly prove that *Bh* ∈ *P*_*h*_. Thus condition (i) of Theorem 4 is satisfied.

In the following we show that condition (ii) of Theorem 4 holds. From (A3),

(22) (Au)(t)=∑s=1TG(t,s)f(s,u(s))≥δ0∑s=1TG(t,s)g(s,u(s))=δ0(Bu)(t).

Then we get *Au* ≥ *δ*_0_ *Bu*, *u* ∈ *P*. By applying Theorem 4, it can be obtained that the operator equation *Au* + *Bu* = *u* has a unique solution *u* ^*^ ∈ *P*_*h*_. Moreover, constructing successively the sequence *u*_*n*_ = *Au*_*n*−1_ + *Bu*_*n*−1_, *n* = 1,2,…, for any initial value *u*_0_ ∈ *P*_*h*_, we have *u*_*n*_ → *u* ^*^ as *n* → *∞*. That is, problem (1)-(2) has a unique positive solution *u* ^*^ ∈ *P*_*h*_. In addition, for any initial value *u*_0_ ∈ *P*_*h*_, constructing successively the sequence

(23) un+1(t)=∑s=1TG(t,s)[f(s,u(s))+g(s,u(s))],iiiiiiiiiiiiiiiiiiiiiiiiiiiiiiiiiiiiiiiiin=0,1,2,…,

we have *u*_*n*_(*t*) → *u* ^*^(*t*) as *n* → *∞*.

The following result can be obtained by Remark 5 and Theorem 4.

**Corollary 9 .**

Assume that

 (A1)′ *f* : *ℤ*_0,*T*+1_ × [0, *∞*)→[0, *∞*) is continuous and increasing with respect to the second variable, *f*(*T*, 0) ≠ 0;
 (A2)′ there exists a constant *γ* ∈ (0, 1) such that *f*(*t*, *λx*) ≥ *λ* ^*γ*^ *f*(*t*, *x*) for *λ* ∈ (0, 1), *t* ∈ *ℤ*_0,*T*+1_, *x* ∈ [0, *∞*).

Then problem

(24) $$\begin{array}{c} -\Delta^{2}u\left(t-1\right)=f\left(t,u\left(t\right)\right),\;t\in Z_{1,T},\\ u\left(0\right)-\beta \Delta u\left(0\right)=0,\;\;u\left(T+1\right)=\alpha u\left(\eta \right) \end{array}$$

has a unique positive solution *u* ^*^ ∈ *P*_*h*_, where *h*(*t*) = *t* + *β*, *t* ∈ *ℤ*_0,*T*+1_. Moreover, for any initial value *u*_0_ ∈ *P*_*h*_, constructing successively the sequence

(25) $$\begin{array}{c} u_{n+1}\left(t\right)=\overset{T}{\underset{s=1}{\sum }}G\left(t,s\right)f\left(s,u_{n}\left(s\right)\right),\;n=0,1,2,\ldots , \end{array}$$

we have *u*_*n*_(*t*) → *u* ^*^(*t*) as *n* → *∞*, where *G*(*t*, *s*) is given as (9).

**Remark 10 .**

In a similar way, we can get the corresponding results for the difference equation (1) subject to boundary conditions

(26) $$\begin{array}{c} u\left(0\right)=\alpha u\left(\eta \right),\;\;u\left(T+1\right)+\beta \nabla u\left(T+1\right)=0, \end{array}$$

which are symmetric to the boundary condition (2).

**Remark 11 .**

The results can also be generalized to discrete *m*-point boundary value problems:

(27) $$\begin{array}{c} -\Delta^{2}u\left(t-1\right)=f\left(t,u\left(t\right)\right)+g\left(t,u\left(t\right)\right),\;t\in Z_{1,T},\\ u\left(0\right)-\beta \Delta u\left(0\right)=0,\;\;u\left(T+1\right)=\overset{m-2}{\underset{i=1}{\sum }}\alpha_{i}u\left(\xi_{i}\right),\\ -\Delta^{2}u\left(t-1\right)=f\left(t,u\left(t\right)\right)+g\left(t,u\left(t\right)\right),\;t\in Z_{1,T},\\ u\left(0\right)=\overset{m-2}{\underset{i=1}{\sum }}\alpha_{i}u\left(\xi_{i}\right),\;\;u\left(T+1\right)+\beta \nabla u\left(T+1\right)=0. \end{array}$$

**Example 12 .**

Consider the following nonlinear discrete problem:

(28) $$\begin{array}{c} -\Delta^{2}y\left(t-1\right)=f\left(t,y\left(t\right)\right)+g\left(t,y\left(t\right)\right),\;t\in Z_{1,9},\\ y\left(0\right)-2\Delta y\left(0\right)=0,\;\;y\left(10\right)=\frac{1}{2}y\left(4\right). \end{array}$$

If we set

(29) $$\begin{array}{c} f\left(t,y\right)=y^{1/4}+t^{4}+\frac{\pi }{2},\;\left(t,y\right)\in Z_{0,10}\times \left[0,\infty \right),\\ g\left(t,y\right)=\arctan y+t^{3},\;\left(t,y\right)\in Z_{0,10}\times \left[0,\infty \right), \end{array}$$

then *f*, *g* : *ℤ*_0,10_ × [0, *∞*)→[0, *∞*) are continuous and increasing with respect to the second variable, *g*(9, 0) ≠ 0, and for *λ* ∈ (0, 1), *t* ∈ *ℤ*_0,10_, *y* ∈ [0, *∞*),

(30) f(t,λy)=λ1/4y1/4+t4+π2≥λ1/4(y1/4+t4+π2)=λ1/4f(t,y),g(t,λy)=arctan⁡(λy)+t3≥λarctany +t3≥λ(arctany+t3)=λg(t,y).

In addition,

(31) f(t,y)=y1/4+t4+π2≥t4+π2≥t3+π2≥12(arctany+t3)=12g(t,y).

Thus, all conditions of Theorem 8 are satisfied and so problem (28) has a unique positive solution in *P*_*t*+2_.

## 3. Positive and Uniqueness of Solutions to BVP (1) and (3)

**Lemma 13 (see [6]).**

If *α* ≠ 1, the nonlocal boundary value problem

(32) $$\begin{array}{c} -\Delta^{2}u\left(t-1\right)=y\left(t\right),\;t\in Z_{1,T},\\ \Delta u\left(0\right)=0,\;\;u\left(T+1\right)=\alpha u\left(\eta \right) \end{array}$$

has a unique solution

(33) $$\begin{array}{c} u\left(t\right)=\overset{T}{\underset{s=1}{\sum }}\hat{G}\left(t,s\right)y\left(s\right), \end{array}$$

where

(34) G^(t,s)={T+1−t+α(t−η)1−α,s<min⁡{t,η},T+1−t+α(t−s)1−α,η≤s<t,T+1−s+α(s−η)1−α,t≤s<η,T+1−s1−α,max⁡{t,η}≤s.

**Lemma 14 .**

For *t* ∈ *ℤ*_0,*T*+1_, *s* ∈ *ℤ*_1,*T*_, the Green function *G*(*t*, *s*) in Lemma 13 has the following property:

 (i) *G*(*t*, *s*) > 0, *t* ∈ *ℤ*_0,*T*+1_, *s* ∈ *ℤ*_1,*T*_;
 (ii) $G(t,s)\leq \hat{r}(s)$, *t* ∈ *ℤ*_0,*T*+1_, *s* ∈ *ℤ*_1,*T*_, where
  (35) $$\begin{array}{c} \hat{r}\left(s\right)=\frac{T+1-s+\alpha T}{1-\alpha }. \end{array}$$

**Proof**

We omit it since it is obvious.

**Theorem 15 .**

Assume that (A1), (A2), and (A3) are satisfied; then the problem (1)–(3) has a unique positive solution ${\hat{u}}^{*}\in P_{h}$, where *h*(*t*) = 1, *t* ∈ *ℤ*_0,*T*+1_. Moreover, for any initial value ${\hat{u}}_{0}\in P_{h}$, constructing successively the sequence

(36) u^n+1(t)=∑s=1TG^(t,s)[f(s,u^n(s))+g(s,u^n(s))],iiiiiiiiiiiiiiiiiiiiiiiiiiiiiiiiiiiiiiiiiiiin=0,1,2,…,

we have ${\hat{u}}_{n}(t)\to {\hat{u}}^{*}(t)$ as *n* → *∞*, where $\hat{G}(t,s)$ is given as (34).

**Proof**

It is similar to the proof of Theorem 8.

The following corollary can be obtained by Remark 5 and Theorem 4.

**Corollary 16 .**

Assume that (A1)′ and (A2)′ are satisfied; then the problem

(37) $$\begin{array}{c} -\Delta^{2}u\left(t-1\right)=f\left(t,u\left(t\right)\right),\;t\in Z_{1,T},\\ \Delta u\left(0\right)=0,\;\;u\left(T+1\right)=\alpha u\left(\eta \right) \end{array}$$

has a unique positive solution ${\hat{u}}^{*}\in P_{h}$, where *h*(*t*) = 1,  *t* ∈ *ℤ*_0,*T*+1_. Moreover, for any initial value ${\hat{u}}_{0}\in P_{h}$, constructing successively the sequence

(38) $$\begin{array}{c} {\hat{u}}_{n+1}\left(t\right)=\overset{T}{\underset{s=1}{\sum }}\hat{G}\left(t,s\right)f\left(s,{\hat{u}}_{n}\left(s\right)\right),\;n=0,1,2,\ldots , \end{array}$$

we have ${\hat{u}}_{n}(t)\to {\hat{u}}^{*}(t)$ as *n* → *∞*, where $\hat{G}(t,s)$ is given as (34).

**Remark 17 .**

In a similar way, we can get the corresponding results for the difference equation (1) subject to boundary conditions

(39) $$\begin{array}{c} u\left(0\right)=\alpha u\left(\eta \right),\;\;\Delta u\left(T+1\right)=0, \end{array}$$

which are symmetric to the boundary condition (3).

**Remark 18 .**

The results can also be generalized to discrete *m*-point boundary value problems:

(40) $$\begin{array}{c} -\Delta^{2}u\left(t-1\right)=f\left(t,u\left(t\right)\right)+g\left(t,u\left(t\right)\right),\;t\in Z_{1,T},\\ \Delta u\left(0\right)=0,\;\;u\left(T+1\right)=\overset{m-2}{\underset{i=1}{\sum }}\alpha_{i}u\left(\xi_{i}\right),\\ -\Delta^{2}u\left(t-1\right)=f\left(t,u\left(t\right)\right)+g\left(t,u\left(t\right)\right),\;t\in Z_{1,T},\\ u\left(0\right)=\overset{m-2}{\underset{i=1}{\sum }}\alpha_{i}u\left(\xi_{i}\right),\;\;\Delta u\left(T+1\right)=0. \end{array}$$

**Example 19 .**

Consider the following nonlinear discrete problem:

(41) $$\begin{array}{c} -\Delta^{2}y\left(t-1\right)=f\left(t,y\left(t\right)\right)+g\left(t,y\left(t\right)\right),\;t\in Z_{1,4},\\ \Delta y\left(0\right)=0,\;\;y\left(5\right)=\frac{3}{5}y\left(3\right). \end{array}$$

If we set

(42) $$\begin{array}{c} f\left(t,y\right)=y^{2/3}+e^{t},\;\left(t,y\right)\in Z_{0,5}\times \left[0,\infty \right),\\ g\left(t,y\right)=\frac{y}{1+y}t^{2}+1,\;\left(t,y\right)\in Z_{0,5}\times \left[0,\infty \right), \end{array}$$

then *f*, *g* : *ℤ*_0,5_ × [0, *∞*)→[0, *∞*) are continuous and increasing with respect to the second variable, *g*(4, 0) ≠ 0, and for *λ* ∈ (0, 1), *t* ∈ *ℤ*_0,5_, *y* ∈ [0, *∞*),

(43) f(t,λy)=λ2/3y2/3+et≥λ2/3(y2/3+et)=λ2/3f(t,y),g(t,λy)=λy1+λyt2+1≥λ(y1+yt2+1)=λg(t,y).

In addition,

(44) f(t,y)≥e0=126·26≥126·(y1+yt2+1)=126g(t,y).

Thus, all conditions of Theorem 15 are satisfied and so problem (41) has a unique positive solution in *P*_1_.
